# Crossing the Line: Factors Associated With Escalating Pornography Use

**DOI:** 10.1177/08862605261462151

**Published:** 2026-06-29

**Authors:** Melissa S. de Roos, Laura Willkomm

**Affiliations:** 1 Erasmus University Rotterdam, The Netherlands

**Keywords:** pornography, offenders/perpetrators, child, sexual abuse, internet and abuse

## Abstract

Child Sexual Abuse Material (CSAM) offending has increased in recent years. Rather than being predominantly driven by an increase in associated sexual interest in children, this may be understood as a pattern of content escalation, from mainstream to extreme or even illegal pornography. This study examined patterns of content escalation in pornography consumption, psychosocial predictors of such escalation and their association with engagement with CSAM in a cross-sectional study with a non-clinical male sample (*N* = 228). Participants reported arousal, viewing frequency, and content escalation across mainstream, extreme and illegal categories. Principal component analysis revealed five distinct content escalation clusters: Dominance/Degradation, Extreme/Harmful, Non-Traditional Niche, Normative and Atypical. While mainstream content exhibited the highest absolute viewing and mean escalation, relative escalation was disproportionately observed in more extreme and niche categories, such as S&M, fetish, teen, group sex and violent content.

## Introduction

The use of Child Sexual Abuse Material (CSAM) has increased substantially over the past decade. The urgency of this issue is highlighted by the record-high number of suspected CSAM URLs reported in 2024 by INHOPE (2025), reflecting a 202% increase in illegal content compared to the previous year. Potential explanations for this exponential growth include evolving supply-side dynamics such as the financial incentives derived from sexual extortion, the use of realistic, AI-generated content (Christensen et al., 2021; INHOPE, 2025), as well as self-generated material shared on messaging platforms (INHOPE, 2025). Importantly, this increase does not necessarily indicate a corresponding rise in underlying sexual deviance. Rather, it appears to be driven by situational factors linked to the increasing accessibility and normalisation of online pornography, which may inadvertently facilitate exposure to extreme, and in some cases illegal, material. Understanding if and how non-illegal pornography use can escalate towards the consumption of extreme or illicit content is therefore essential.

The motivations driving the consumption of CSAM are complex and varied, challenging the prevailing assumption that all CSAM consumers have a pre-existing sexual preference for child material. Alternative explanations emphasise a distinct pathway: the escalation of mainstream pornography consumption (Quayle & Taylor, 2003; Sharpe & Mead, 2021). This pathway is driven by a progressive need for increasingly extreme pornographic content to achieve the same level of arousal. The underlying mechanism is desensitisation: prolonged exposure to mainstream pornography reduces arousal responsiveness, prompting individuals to seek greater novelty and intensity of content to reach the same level of arousal (Koukounas & Over, 1993; O’Donohue & Geer, 1985; Rimer & Holt, 2023). Consequently, diminished arousal to readily available, non-illegal pornography can lead to extended search behaviour and exposure to progressively more extreme content, in some cases resulting in the consumption of CSAM. In such instances, the primary motivation may shift from a specific sexual interest towards thrill-seeking behaviour and the pursuit of content extremity; individuals may initially be drawn by the shock value and novelty, rather than the depiction of children per se. This hypothesised escalation pathway represents a critical yet understudied mechanism that may explain how some individuals progress towards illegal content. Despite recognition of this risk, substantial uncertainty remains regarding how this process manifests in non-clinical populations, particularly how engagement with various non-CSAM genres and themes relate to patterns of content escalation. To address this gap, this study examines individuals’ pornography consumption through the clustering of different content types.

### Measuring Escalation

Pornography research has long struggled to establish a consistent taxonomy capable of classifying the vast heterogeneity of available material (Ballester-Arnal et al., 2022; Hald & Štulhofer, 2016). Classification approaches vary considerably, from broad distinctions between normophilic (e.g. vaginal or oral sex) and paraphilic content (e.g. violent, coercive sex, fetishistic; Ballester-Arnal et al., 2022) to groupings based on user demographics (Hald & Štulhofer, 2016). Although some favour granular operational classifications focusing on content complexity (e.g. performer characteristics, sexual vs. non-sexual behaviours and setting; Kohut et al., 2024), a thematic framework based on explicit content categories consumed is often more practical for studying escalation. Focusing on themes prevalent across major content platforms provides a direct means of understanding how users navigate and progress through readily available, non-illegal material towards illegal CSAM. Therefore, a clearer empirical framework for categorising and analysing how content types co-occur in user consumption patterns is essential.

Existing literature often simplifies escalation by focusing solely on viewing frequency, implicitly assuming individuals only consume what they find highly arousing. However, emerging evidence challenges this assumption, revealing a critical disconnect between arousal and viewing behaviour. Paul (2009), for instance, found that the most frequently viewed content is not necessarily the most arousing, particularly as content becomes more extreme. For example, men ranked “barely legal” pornography among their five most arousing categories, yet it did not appear among their five most frequently viewed categories (Paul, 2009). Conversely, women reported lower arousal towards content depicting ejaculations but still included it among their most frequently viewed categories (Paul, 2009). These findings suggest that desensitisation, which drives the search for novelty and extremity, compels users to engage with content despite diminished arousal. To accurately capture the desensitisation mechanism underpinning escalation towards illegal CSAM, research must integrate frequency, content type and subjective arousal when mapping consumption patterns. These findings are consistent with desensitisation-based accounts of content escalation (Knack et al., 2020), in which repeated exposure reduces arousal over time (Koukounas & Over, 1993; O’Donohue & Geer, 1985), resulting in a decoupling between subjective arousal and consumption behaviour. As such, continued engagement with less arousing content provides a theoretically grounded indicator of escalation-related processes.

### Psychosocial Predictors of Escalation

Increased pornography use may precipitate content escalation, a process that fundamentally resembles tolerance, defined by the need for novel or more sexual stimuli to achieve the same level of arousal (Lewczuk et al., 2022; O’Donohue & Geer, 1985; O’Donohue & Plaud, 1991). This tolerance towards mainstream material can lead users to shift from normophilic to paraphilic content in pursuit of heightened stimulation (Krahé et al., 2011). Such progression towards extreme material is critical for understanding the consumption of illegal content such as CSAM, suggesting that some individuals consume CSAM primarily for its extremity, rather than due to a specific sexual interest in children (Merdian et al., 2018; Quayle & Taylor, 2003); a distinction with substantial implications for intervention and prevention strategies.

Sexual sensation-seeking (SSS), defined as the pursuit of novel or risky sexual stimuli (Kalichman et al., 1994) has been identified as a key psychological factor underlying this escalation process. The central hypothesis is that individuals high in SSS are more likely to seek extreme material to satisfy their heightened desire for sexual intensity (Chen et al., 2018; Sinković et al., 2013), a drive that mirrors tolerance-driven desensitisation (Bocci Benucci et al., 2024). Although findings on the relationship between SSS and pornography use are mixed (Esplin et al., 2024; Sinković et al., 2013), sensation-seeking tendencies align closely with mechanisms of desensitisation and novelty-seeking that fuel content escalation. For example, increased viewing frequency was found to predict a shift towards more hardcore content and greater openness to paraphilic and fetish content (e.g. rape or hebephilic themes; Blinka et al., 2022). Collectively, these findings indicate that SSS contributes not merely to the frequency of pornography use, but to the qualitative nature of the material sought, making it a key predictor in the escalation pathway.

A second key predictor of escalation is Problematic pornography use (PPU), defined as a loss of control over consumption and continued use despite adverse consequences (Gibbons et al., 2020). PPU is intrinsically linked to the consumption of increasingly extreme material, with genre escalation identified as a relevant dimension of PPU severity (Ince et al., 2024; Lewczuk et al., 2021). These findings suggest that the behavioural pattern of PPU drives individuals to seek progressively more diverse and extreme content. Furthermore, PPU appears to be closely related to SSS, indicating a potential interactive pathway to escalation. Sensation-seeking tendencies are a recognised vulnerability factor for PPU (Hegbe et al., 2021), and several studies report positive associations between the two constructs (Bőthe et al., 2019). This relationship implies that the drive for novelty and heightened stimulation (SSS) may contribute to the compulsive, uncontrolled consumption that defines PPU. Together, these processes likely operate synergistically, with SSS amplifying the motivational drive for novelty, and PPU reinforcing the behavioural pattern of compulsive use, thereby accelerating the progression toward increasingly extreme and, in some cases, illegal material.

The third factor relevant to the development of problematic escalation patterns is the endorsement of myths about child sexual abuse (CSA). These myths function as offence-supportive cognitions, enabling individuals to rationalise and normalise abusive behaviour (Collings, 1997). Categories of these myths include the minimisation of harm, denial of the extent of CSA, diffusion of perpetrator responsibility, and reliance on perpetrator stereotypes (Cromer & Goldsmith, 2010). Endorsing such beliefs reduces cognitive dissonance and facilitates the perception of children as sexual beings, thereby normalising the consumption of related content. These cognitive processes are evident in pornography consumption, as individuals who endorse CSA myths are more likely to escalate their viewing patterns toward extreme material (Osbourne & Christensen, 2020). This mechanism parallels the influence of rape myths on the consumption of violent pornography (Bhuptani et al., 2024). Critically, at the extreme end of the escalation spectrum, these beliefs provide internal justification to consume illegal material. Complementing these cognitive rationalisations, the perceived or actual acceptance of CSAM viewing within one’s peer group may further reduce inhibition and reinforce the likelihood of consuming illegal material (Schonert-Reichl, 1999).

### The Present Study

The present study addresses a gap in understanding early stages of escalation-related processes from mainstream pornography consumption towards extreme or illegal content. Previous research has often operationalised escalation merely as a symptom of PPU (Ince et al., 2024). Our approach focuses on thematic escalation and treats this as an independent phenomenon. Moving beyond frequency metric, we investigate how escalation clusters across different thematic content types, conceptualising escalation a behavioural pattern characterised by the continued viewing of content despite low or diminished arousal (Paul, 2009), consistent with desensitisation-based accounts of habituation to sexual stimuli (Knack et al., 2020). Importantly, this approach does not assume a single underlying motivation; rather, it captures a pattern of engagement that may arise from multiple pathways (e.g. curiosity, openness to experience), but which nevertheless reflects the behavioural conditions under which desensitisation and progression across content types may occur. We note that a definitive test of escalation would require longitudinal data to examine changes over time; our cross-sectional design allows us to characterise patterns of content engagement that could reflect early stages of escalation-related processes.

Additionally, systematically examine the psychosocial correlates of these distinct escalation patterns within a non-clinical population. Although factors like SSS, PPU and the endorsement of CSA myths have been linked to general pornography consumption, their specific relationships with content escalation patterns in the general population remains unclear. This focus enables generalisation beyond clinical populations.

To this end, our research questions are:

**R1:** Across what content do people escalate in their pornography consumption?
**H1:** We expect that escalation will occur across multiple content categories, with the highest absolute consumption occurring in normophilic/mainstream categories and the most significant relative increase (escalation) in paraphilic/extreme content.
**R2:** Do patterns of escalation cluster in terms of content?
**H2:** Patterns of reported escalation will cluster into distinct groups that align along a continuum of content extremity (e.g. from mainstream-only escalation to taboo/illegal content escalation)
**R3:** Which psychosocial factors (SSS, PPU, CSA myths, and peer attitudes towards CSAM) are related to problematic patterns of content escalation?
**H3:** SSS and PPU will be positively related to escalation clusters across the entire continuum (mainstream to extreme) (H3a), and CSA Myths and Peer Attitudes towards CSAM will be uniquely and most strongly related to the escalation clusters aligned with taboo and illegal content (H3b).
**R4:** How does escalating pornography use in terms of content relate to engaging with CSAM?
**H4:** We expect the escalation cluster showing the highest consumption of taboo content will demonstrate the strongest positive association with engagement with illegal CSAM.

## Methods

### Participants

Participants were recruited from the university research participant platform and through social media channels. Those who participated through the university received 0.5 research credits for their participation. Our inclusion criteria were men between the ages of 18 and 35 who viewed pornography. Initially, 395 participants clicked the link to the survey. Nineteen participants were removed for falling outside the age range and 60 for indicating their gender as anything other than man, and three for not providing informed consent. A further 85 completed less than 75% of the survey and were deleted, resulting in a final sample of *N* = 228 (*M*_age_ = 22.65, *SD*_age_ = 3.16). This sample comprised predominantly single (54%), heterosexual (82%), students (61%) who identified as white (76%). These three percentages are based on the participants who answered these questions, but each question had 19% missing data. This likely occurred because participants were not clearly prompted to complete the final page of demographic questions; all percentages for these items are reported as valid percentages among respondents who provided data.

### Materials

#### SSS Scale

The SSS scale (Kalichman et al., 1994), is a questionnaire comprising ten items to assess the need for novel sexual experiences (e.g. “I feel like exploring my sexuality”). Participants indicate how well each statement describes them on a four-point Likert scale (1 = *not at all like me*, 4 = *very much like me*). Item scores were summed, with higher total scores indicating greater sensation-seeking. The internal reliability was good (α = .82).

#### Problematic Pornography Consumption Scale

The Problematic Pornography Consumption Scale (PPCS; Bőthe et al., 2018) was used to measure the degree of participants’ problematic pornography consumption. The questionnaire consists of 18 items and is based on the six-component addiction model by Griffith (2005). It contains the following subscales: salience (e.g. “I thought how good it would be to watch porn”), mood modification (e.g. “watching porn got rid of my negative feelings”), conflict (e.g. “watching porn prevented me from bringing out the best in me”), tolerance (e.g. “I felt that I needed more and more porn in order to satisfy my needs”), relapse (e.g. “when I vowed not to watch porn anymore, I could only do it for a short period of time”), and withdrawal (e.g. “I became agitated when I was unable to watch porn”). Participants indicated how often each statement was true for them on a seven-point Likert scale ranging (1 = *never*, 7 = *all the time*). Scores were determined for the six subscales by summing the items of each scale with higher scores indicating a more problematic consumption of pornography. The internal reliability for the scale was excellent (α = .94).

#### CSA Myths Scale

CSA Myth Scale, a scale comprising 15 statements about, was administered to measure the acceptance of CSA stereotypes (Collings, 1997). The self-reported questionnaire contains 15 statements about CSA. Participants were asked to indicate their agreement with these statements on a five-point Likert scale (1 = *strongly agree*, 5 = *strongly disagree*). Example statements include: “Sexual contact with an adult can contribute favourably to a child’s subsequent psycho-sexual development” and “Adolescent girls who wear very revealing clothing are asking to be sexually abused.” Individual item scores were summed, with higher scores indicating a greater acceptance of CSA myths. The internal reliability was good (α = .84).

#### Peer Acceptance of CSAM

Peer acceptance of CSAM was measured by asking participants to what extent they agreed with the following two statements: “Some of my friends and acquaintances think sex with children is ok” and “Some of my friends have watched child pornography on the Internet,” both scored on a five-point Likert scale (1 = *strongly agree*, 5 = *strongly disagree*). Both scores were summed, with higher scores indicating a higher level of peer acceptance. Internal reliability was excellent (α = .93).

#### Pornography Consumption Questionnaire

Subsequently, participants completed the pornography consumption questionnaire (based on Hald, 2006). The self-reported questionnaire includes 10 questions, assessing both general patterns of pornography use and specific pornographic interests. The general items capture aspects such as frequency and onset of use (e.g. “When did you last use pornography?” or “How old were you when you first used pornography?”), while the content-specific items assess levels of arousal and interest across a range of pornographic categories. These categories include a wide spectrum of sexual themes, for example, “oral sex,” “anal penetration,” “rape,” or “teens.”

#### CSAM Use

Lastly, we asked participants about their experiences with CSAM. They were first asked whether they had ever encountered pornographic material depicting individuals under the age of 18 or children. If participants responded affirmatively, they were subsequently asked to indicate their emotional reactions upon the discovery of such material, their thought on the content, whether they discussed the experience with others, whether they would consider viewing similar content again, and whether they sought help after the encountering of such material. Responses were coded on an ordinal scale, e.g. participants who indicated that they sought help (“*yes*”) were assigned a value of 1, whereas those who had not (“*no*”) were coded as 0.

### Procedure

This study received ethical approval from the university institutional review board. Participants were directed to the study in Qualtrics through an anonymous link. After providing informed consent, participants were presented with the questionnaires in randomised order. They had the option to skip any questions they felt not comfortable answering and could take breaks whilst completing the survey. Finally, they were asked demographic questions and presented with a debrief form that included contact information for various support organisations for people concerned about their pornography use.

### Analyses

To operationalise escalation within each pornography category, we created an index that reflects the extent to which participants reported viewing a category despite not finding it highly arousing. This was done by multiplying the reported frequency of viewing the category (0 = *never*, 4 = *all the time*) by a reverse-coded arousal score (4 – arousal rating, where 0 = *not at all arousing*, 4 = *very arousing*). The resulting escalation score therefore increases when participants indicate relatively high use of a category that they rate as less arousing. Conceptually, this captures the degree of consumption that extends beyond an individual’s stated sexual interests, which we interpret as indicative of escalation. Scores range from 0 to 16 with higher scores indicating greater escalation. This operationalisation is grounded in the premise that escalation is reflected not only in exposure to different content types, but in continued engagement in the absence of strong subjective arousal, which is consistent with desensitisation processes. Although such patterns may also reflect individual differences in curiosity, openness, or exploratory tendencies, repeated exposure to content that is not highly arousing may nevertheless contribute to habituation over time. Further, while our cross-sectional design does not measure escalation longitudinally, this index provides a meaningful behavioural indicator of engagement with less arousing content, which may reflect early-stage escalation process.

We examined frequencies of arousal, viewing and escalation per pornography category and descriptive statistics of all predictor variables. Following this, we conducted a principal components factor analysis to examine how escalation clusters across content. Based on the results, five factors were identified and participants’ individual scores for each factor were computed. These scores were then used in a series of multiple regressions to examine whether escalation in that factor could be predicted from the independent variables in our study. All predictors were assessed for multicollinearity. Variance inflation factor (VIF) values ranged from 1.29 to 3.42, and Tolerance values ranged from 0.29 to 0.78, indicating no problematic multicollinearity across analyses.

## Results

Most participants reported first viewing pornography between ages 9 to 12 years (41%) or 13 to 16 (43%). Regarding the context of pornography use, most participants reported engaging in sexual activity alone (masturbation) often or always (93.3%), whereas only a small proportion reported sexual activity with a partner often or always (3.1%). Although 75% perceived pornography as unrealistic, 80% considered its use acceptable. Weekly viewing was typically under 1 hr (45%) or between 1 and 3 hr (35%), with the majority reporting frequently or always masturbating during consumption (92%). Regarding extreme content, 49% indicated they had searched for pornography in specific age-related categories, most commonly teen content (27%). Only two participants indicated they had searched for content depicting children, although 6% had come across such content and 5% were unsure. Conversely, 12% reported encountering content depicting individuals under eighteen and 39% were not sure if they had. Among those who had (maybe) viewed these categories, 10% searched for it again at a later date, with 7% reporting arousal and 10% reporting curiosity. Overall, 10% indicated they had watched pornography that was illegal in their country of residence, whilst 21% were not sure.

Percentages of participants who indicated some arousal, who had ever viewed each category, and the mean and standard deviation of viewing content more frequently than their arousal warranted (Content Escalation) are displayed in Table 1. Consistent with H1, absolute arousal and viewing were highest for mainstream content. Relative content escalation, however, was disproportionately observed in more extreme categories such as S&M/bondage, fetish, teen, group sex, and violent sex. The lowest arousal, viewing and content escalation scores were observed in the most extreme categories, reflecting the relative rarity of engagement with illegal material.

**Table 1.** Frequencies in Percentages of Arousal, Viewing and Escalation Across all Pornography Content Types.

Type	Arousal	Viewing	Escalation
Vaginal penetration	91.1	91.5	2.78 (2.50)
Anal penetration	72.9	78.1	2.05 (1.90)
Hentai	31.6	40.8	0.98 (1.70)
Oral sex	90.7	91.5	3.16 (2.94)
Rape	15.1	20.2	0.45 (1.14)
S&M/Bondage	39.3	42.9	1.19 (1.70)
Sex with animals	4.0	4.5	0.13 (0.65)
Sex with people <18	5.3	11.7	0.18 (1.25)
Sex with children	2.2	3.6	0.04 (0.39)
Snuff	2.7	3.6	0.08 (0.57)
Heterosexual content	92.6	93.3	2.47 (2.62)
Lesbian content	68	78.6	1.52 (1.67)
Gay male content	24.4	27.4	0.50 (1.30)
Fetish	46.7	48.7	1.57 (2.10)
Teen	54.7	55.5	1.83 (2.21)
Group sex	67.1	70.1	2.16 (2.23)
Violent sex	36.6	35.4	1.20 (1.77)
Bukkake	20.4	25.6	0.59 (1.41)
Sex involving urine	15.1	15.6	0.51 (1.29)
Sex involving faeces	3.1	4.0	0.08 (0.49)

To examine how escalation clusters across content types (H2), we conducted a principal component analysis with Promax rotation and Kaiser normalisation. Inspection of the scree plot indicated a five-factor solution, explaining 50.27% of total variance. The Principal Component Analysis was then re-run specifying five factors. The pattern matrix is displayed in Table 2.

**Table 2.** Factor Loadings of Principal Component Analysis of Escalation Across Pornography Content Types.

Variable	Dominance degradation	Extreme harmful	Non-traditional Niche	Normative	Atypical
Vaginal penetration				.**83**	
Anal penetration	.30		.30		.**53**
Hentai		−.19	.**79**		
Oral sex			−.11	.**52**	.24
Rape	.**51**	.17	.30		
S&M/Bondage	.**59**	.23			
Sex with animals		.15	.**63**		
Sex with people <18	−.23	.**41**	.41	.16	
Sex with children	−.14	.**82**	.19		
Snuff	.15	.**77**	−.12		.22
Heterosexual content				.**83**	
Lesbian content		.22		.17	.**64**
Gay male content	.12		.**54**		
Fetish	.**67**	−.12			
Teen	.**32**		.19		
Group sex	.**63**	−.11	−.21	.20	.12
Violent sex	.**66**			−.20	
Bukkake	.**50**		.12	.10	−.48
Sex involving urine	.24	.27			−.62
Sex involving faeces		.**78**	−.19		

*Note.* Loadings <.10 repressed. Bolded loadings represent allocation to factor.

From this, the five identified factors are: (1) Dominance/Degradation, (2) Extreme/Harmful, (3) Non-Traditional Niche, (4) Normative and (5) Atypical Escalation. Oblique rotation confirmed moderate intercorrelations between factors (−.27–.28), supporting the use of the Promax rotation. The strongest positive correlation was observed between Extreme/Harmful and Non-Traditional Niche Escalation (*r* = .28), indicating shared tendencies towards highly non-mainstream content. The strongest negative correlation was found between Non-Traditional Niche and Atypical Escalation (*r* = −.27), suggesting these two forms of content escalation are weakly and inversely related. These findings broadly support H2, demonstrating that escalation patterns cluster along a continuum of content extremity, ranging from mainstream to extreme and idiosyncratic categories.

Following identification of the five distinct escalation dimensions, individual factor scores were computed for each participant using the regression method in SPSS. This generated standardised scores (mean μ = 0, standard deviation σ = 1) that account for both the unique contribution of each content category and the correlations among factors. The resulting factor scores were then used as dependent variables in subsequent analyses. Following this, we looked at the descriptive statistics of our predictor variables, and their correlations with the five identified factors (Table 3). Consistent with H3a, SSS and PPU were consistently and most strongly correlated with Dominance/Degradation, indicating that motivational and behavioural factors drive content escalation across moderate-extremity content. Conversely, CSA myths and CSAM peer acceptance were uniquely correlated with Extreme/Harmful, supporting H3b and suggesting that cognitive and social mechanisms drive escalation towards the most extreme content.

**Table 3.** Descriptive Statistics of Predictor Variables and Correlations with Five Identified Factors.

Variable	*M*	*SD*	1	2	3	4	5
SSS	22.63	5.84	.37***	.24***	.12	−.09	−.19**
CSA Myths	23.43	7.41	.02	.22**	.10	−.07	−.09
CSAM Peer acceptance	25.45	10.34	.07	.20**	.03	.05	.01
PPU							
Salience	4.60	4.12	.46***	.17*	.16*	.02	−.24***
Mood	5.59	4.16	.41***	.13	.15*	.03	−.17*
Conflict	4.39	4.37	.24***	.08	.02	.02	−.12
Tolerance	3.62	4.00	.43***	.20**	.12	.05	−.19**
Relapse	4.69	4.97	.21**	<.01	.01	.04	.03
Withdrawal	2.28	3.33	.34***	.19**	.07	.08	−.19*
Total	25.17	20.42	.42***	.15*	.10	.05	−.17*

*Note.* 1 = *Dominance/Degradation*, 2 = *Extreme/Harmful*, 3 = *Non-traditional Niche*, 4 = *Normative*, 5 = *Atypical*. SSS = Sexual sensation-seeking; CSA = Child Sexual Abuse; CSAM = Child Sexual Abuse Material; PPU = Problematic pornography use.

*** *p* < .001. **p < .01. *p < .05.

We then conducted a series of multiple regressions with the five-factor scores as dependent variable, and SSS, CSA myth endorsement, CSAM peer acceptance and the six subscales of PPU as predictors. The model for Dominance/Degradation was significant (*F*[9, 212] = 9.86, *p* < .001) explaining 27.3% of variance (Table 4). SSS and tolerance were significant, positive predictors whereas CSA myths was a significant negative predictor, further supporting H3a and demonstrating that motivational and tolerance-related processes predict escalation in moderate-extremity content.

**Table 4.** Multiple Regression Analyses Predicting Escalation across Dominance/Degradation, Extreme/Harmful and Atypical Content.

Variable	Dominance/Degradation				Extreme/Harmful				Atypical			
	*B*	*SE*	beta	*t*	*B*	*SE*	beta	*t*	*B*	*SE*	beta	*t*
SSS	0.04	0.01	0.21	3.21**	0.02	0.01	0.13	1.77	−0.02	0.01	−0.11	−1.44
CSA Myths	−0.02	0.01	−0.16	−2.21*	0.02	0.01	0.13	1.64	−0.01	0.01	−0.06	−0.75
CSAM Peer	0.01	0.01	0.07	1.10	0.01	0.01	0.10	1.35	0.01	0.01	0.07	0.88
Salience	0.04	0.03	0.16	1.45	0.01	0.03	0.03	0.23	−0.04	0.03	−0.18	−1.43
Tolerance	0.06	0.02	0.24	2.46*	0.03	0.03	0.11	1.07	−0.01	0.03	−0.02	−0.22
Withdrawal	−0.01	0.03	−0.03	−0.27	0.06	0.03	0.18	1.76	−0.03	0.03	−0.11	−1.07
Mood	0.05	0.02	0.19	1.97	−0.01	0.03	−0.02	−0.20	0.00	0.03	0.02	0.17
Conflict	−0.02	0.02	−0.10	−1.03	0.00	0.02	0.00	0.04	−0.03	0.02	−0.15	−1.41
Relapse	0.00	0.02	0.02	0.26	−0.04	0.02	−0.20	−2.17*	0.06	0.02	0.28	3.00*
Model fit	*F* (9, 212) = 9.86, *p* < .001				*F* (9, 212) = 3.83, *p* < .001				*F* (9, 212) = 2.71, *p* = .002			
Adjusted *R*^2^	.273				.107				.080			

*Note.* SSS = Sexual sensation-seeking; CSA = Child Sexual Abuse; CSAM = Child Sexual Abuse Material.

** *p* < .01. *p < .05.

The model for factor 2, Extreme/Harmful, was also significant (*F*[9, 212] = 3.83, *p* < .001) but explained only 10.7% of variance, and only the Relapse subscale of PPU entered the model as a negative predictor (Table 4). This provides partial support for H3b, suggesting that extreme content escalation is less consistently explained by the psychosocial factors included, but relapse tendencies may play a role.

The models for Non-Traditional Niche and Normative content escalation were not significant. For factor 5, Atypical content escalation, the model was significant (*F*[9, 212] = 2.71, *p* = .002) but explained only 8% of variance, and only the Relapse subscale of problematic pornography consumption was a significant predictor (Table 4).

Finally, we conducted Pearson correlations to examine how content escalation across the various factors related to coming across and looking for CSAM. Results are displayed in Table 5. Non-Traditional Niche was the only factor significantly associated with all measured indicators of CSAM engagement. Other factors showed either weaker or nonsignificant associations, indicating that non-mainstream, niche escalation is most relevant for predicting engagement with illegal material.

**Table 5.** Pearson Correlations Between Five Factors and Looking for/Coming Across Child Sexual Abuse Material.

	Searched for		Came across		Ever watched illegal
Factor	Children	Teenagers	Children	<18	
Dominance/Degradation	.08	.27**	.04	.15	.13
Extreme/Harmful	−.07	.17	.08	.01	.03
Non-traditional Niche	.27**	.23*	.33***	.25**	.17*
Normative	.12	−.18	<.01	.07	−.11
Atypical	.02	−.05	−.12	−.09	−.02

*** *p* < .001. **p < .01. *p < .05.

## Discussion

This study examined patterns of content escalation in pornography consumption and their relation to engagement with CSAM in a non-clinical sample. Overall, escalation was observed across all content types, though its magnitude was low. Five distinct escalation content clusters emerged: (1) Dominance/Degradation, (2) Extreme/Harmful, (3) Non-traditional Niche, (4) Normative and (5) Atypical escalation. Importantly, psychosocial variables were cluster-specific: SSS and PPU were more strongly related to Dominance/Degradation content escalation, whereas CSA (peer) myths showed correlations with Extreme/Harmful content escalation. Contrary to our prediction only the Non-Traditional Niche content escalation was reliably associated with engagement of CSAM, suggesting that specialised, idiosyncratic content consumption may be a more sensitive indicator of risk than general escalation toward extreme material. These findings underscore the multidimensional and non-linear nature of pornography content escalation and highlight distinct psychological and social mechanisms underpinning different consumption trajectories. Although we describe patterns of content use that are consistent with hypothesised escalation pathways, these pathways remain conceptual in the present cross-sectional data and should be tested in future longitudinal research.

### Patterns of Escalation

Consistent with prior research (Ballester-Arnal et al., 2022), mainstream categories (e.g. vaginal and oral sex) demonstrated the highest absolute viewing frequencies and mean escalation scores, reflecting the widespread accessibility and consumption of these forms of content. However, when considering relative content escalation, or the proportion of escalation relative to the low baseline arousal and viewing of the category, more extreme and niche categories such as S&M/Bondage, fetish, teen, group sex, and violent sex showed disproportionately greater content escalation. This pattern highlights the coexistence of two distinct forms of two qualitatively distinct escalation processes: one characterised by high-frequency engagement with mainstream content, driven primarily by availability and habitual use, and another reflecting a proportional, high-severity shift toward extreme or non-traditional material, representing a meaningful qualitative change in content interest.

Importantly, even though the absolute engagement with illegal categories (e.g. Sex with children, Snuff, Sex with animals) was minimal, a subset of participants repeatedly sought out these materials despite negative emotional reactions, suggesting there may be a low-frequency, high-risk pathway with significant public health and safety implications. Together, these findings emphasise that content escalation is multidimensional and cannot be fully understood through consumption volume alone; proportional engagement with extreme content captures a critical dimension of risk and potential harm.

Our factor analysis revealed five clusters representing distinct content escalation pathways, further emphasising the heterogeneity of content escalation. The Dominance/Degradation factor captures escalation towards content involving submission and hierarchical sexual scripts, reflecting novelty-driven escalation among those habituated to mainstream content. Conversely, the Extreme/Harmful factor encompasses ethically and legally concerning content, though escalation here was not linked with CSAM involvement. The Normative cluster appears to reflect general habituation, characterised by repeated exposure to high-volume mainstream content (Hald et al., 2014). The Non-Traditional Niche and Atypical clusters suggest branching, specialised content escalation pathways, capturing engagement with niche, paraphilic, or idiosyncratic content rather than generalised sexual compulsivity. Notably, inclusion of Gay Male content in the Non-Traditional Niche factor reflects statistical rarity in a predominantly heterosexual male sample, rather than any deviance, underscoring the need to interpret clusters within demographic context. Little is known about viewing content outside one’s sexual orientation, but this might be an important avenue for future research.

Overall, these clusters suggest that content escalation is not strictly linear, but multidimensional. Although a continuum of severity exists, from mainstream to Dominance/Degradation and Extreme/Harmful, Non-Traditional Niche and Atypical patterns highlight that not all content escalation follows the more widely studied routes of dominance or direct harm, echoing research emphasising the diversity of sexual interests and the complex interplay between novelty, taboo, and individual differences in arousal (Seto, 2012). Such findings reinforce the need for nuanced frameworks that move beyond linear models of escalation and recognise the multidimensionality of sexual content engagement.

The factor structure also aligns with prior research indicating that content characteristics beyond simple extremity shape engagement (Kohut et al., 2024; Sharpe & Mead, 2021). The positive association between the Non-Traditional Niche and the Extreme/Harmful clusters suggest a potentially generalised drive towards non-normative content, where individuals with an interest in one set of unconventional content are also susceptible to an interest in the most severe material (Hald & Štulhofer, 2016). Conversely, the negative relationship between the Non-Traditional Niche and Atypical Escalation might indicate distinct psychological mechanisms underlying these two forms of non-mainstream consumption, but research into such content remains limited (Park et al., 2022). An intense interest in a specific, specialised niche appears to be inversely related to the characteristics that define the more generalised Atypical pattern, reinforcing that interest can branch into specialised, independent routes (Paul, 2009)

### Psychosocial Mechanisms of Escalation

Our analyses highlight cluster-specific psychosocial factors, suggesting that distinct mechanisms drive different forms of content escalation. Dominance/Degradation escalation was associated with SSS and tolerance, indicating that individuals motivated by novel sexual experiences and habituated to mainstream content may be more likely to engage with power- or submission-themed pornography, even when arousal is low. This aligns with prior research linking SSS to arousal for degradation and humiliation themes (Brown et al., 2023). These factors were specific to this content type, highlighting that content escalation in the Dominance/Degradation cluster is distinct from other clusters. Interestingly, higher endorsement of CSA myths was associated with lower likelihood of content escalation in this factor, suggesting that power-dynamics content may operate independently of offence-supportive cognitions.

In contrast, Extreme/Harmful content escalation was minimally associated with the measured variables, with relapse emerging as a negative predictor. This pattern may reflect that individuals escalating in highly extreme content may not perceive their consumption as problematic, and thus are less likely to attempt to reduce consumption. Despite correlations between CSA myths, peer acceptance, and Extreme/Harmful content escalation, these effects did not reach significance in regression analyses, suggesting overlapping variance or limited power. The weak predictive power of this model implies that other factors, such as intrinsic motivation, broader sensation- or thrill-seeking, may drive escalation towards extreme material.

Atypical content escalation, associated with relapse, might capture compulsive or difficult-to-control use, particularly towards idiosyncratic or niche content, consistent with prior research (Camilleri et al., 2021). Conversely, content escalation in the Normative and Non-Traditional Niche clusters was largely independent of measured psychosocial variables, highlighting the potential role of situational factors, such as ease of access, repeated exposure, or individual preference, rather than stable traits.

These results collectively illustrate that content escalation is a multidimensional process with multiple interacting mechanisms: novelty and habituation drive some forms, cognitive rationalisations others, and situational factors yet others, which aligns with prior research differentiating between individual differences and situational factors (Bocci Benucci et al., 2024; Hald et al., 2014). Understanding content escalation thus requires moving beyond one-size-fits-all models and considering the distinct psychological drivers of different consumption patterns.

### Non-Traditional Niche Escalation and CSAM Risk

The strongest link to CSAM engagement emerged in the Non-Traditional Niche content escalation factor, which primarily loads on Hentai, sexualised non-human content, and Gay Male content. As previously discussed, in this predominantly heterosexual male sample, the inclusion of Gay Male content reflects statistically less common consumption rather than deviance per se, highlighting the specialised and non-mainstream nature of this cluster. This factor exhibited consistent correlations with participants’ self-reported behaviours, including searching for, coming across, and watching illegal material. These results suggest that consumption of highly specialised, unconventional, or fantastical content may serve as a more sensitive indicator of potential CSAM engagement than pursuit of aggressive or extreme material alone (Knack et al., 2020; Quayle & Taylor, 2003; Seigfried-Spellar & Rogers, 2013). This pattern is consistent with prior research demonstrating frequent associations between CSAM consumption and engagement with other forms of deviant or non-traditional pornography (Endrass et al., 2009; Seigfried-Spellar & Rogers, 2013). Such patterns indicate that individuals engaging with CSAM may exhibit broader proclivities toward non-traditional sexual content, reflecting fluidity across sexual interests.

One plausible mechanism underlying the observed association is a boundary‑crossing psychological drive. Individuals on the Non‑Traditional Niche pathway may be attracted to sexual content that transcends conventional social and biological scripts, such as stylised Hentai, non‑human imagery, or content featuring ambiguous age‑cues or non‑consensual scenarios, making them more inclined to explore illegal material than individuals following more generalised content escalation patterns, such as Dominance/Degradation (Steel et al., 2021). Research shows that consumers of Hentai exhibit distinct affective and cognitive patterns compared with typical pornography users (Park et al., 2022). Moreover, theoretical and empirical work indicates that Hentai may act as a gateway medium, normalising sexual interest in child‑coded characters or unconventional sexual objects and thereby lowering barriers to illegal consumption (Dines & Sanchez, 2023). A further possibility is that engagement with stylised or cartoonised depictions of minors or non‑human sexual objects may represent an early‑stage paraphilic trajectory, in which content escalation follows specialised rather than purely linear patterns (Christensen & Vickery, 2023). This mechanism aligns with documented overlap between users of CSAM and wider collections of non‑traditional erotic content (Endrass et al., 2009).

Other clusters provide further nuance. Dominance/Degradation content escalation showed a modest link only to searches for teen content, consistent with its focus on power and aggression rather than explicit illegality. This could reflect the perception that younger individuals are easier to dominate than adults (Wall, 2025). Importantly, this pattern may not reflect an interest in children or the taboo/illegal, as may be captured instead by the Extreme/Harmful content escalation, but rather could represent an extension of interest in dominance and submission within a sexual context. This suggests that individuals showing content escalation in this cluster may explore boundary-pushing legal-adult or near-minor content without necessarily engaging in illegal material. In contrast, the Extreme/Harmful content escalation, despite including sexualised depictions of children, was not significantly associated with CSAM engagement. Whilst this finding seems counterintuitive, this finding relates specifically to content escalation (viewing more than arousal warrants), not viewing in general. Individuals may engage with content in this cluster in proportion to their arousal, meaning content escalation scores remain low even when exposure occurs.

Overall, these findings challenge linear models assuming progression toward extreme content predicts CSAM use. Instead, they highlight Non-Traditional Niche content escalation, characterised by highly specialised, boundary-crossing, and non-mainstream consumption, as the clearest empirically observed correlate of CSAM engagement in the present data. These results suggest that research, policy, and prevention initiatives may benefit from considering the psychological and behavioural mechanisms driving specialised content escalation, rather than relying solely on exposure to extreme or aggressive content as an indicator of risk.

### Implications

The present findings contribute to the conceptualisation of pornography content escalation as multidimensional, with qualitatively distinct mechanisms. Whilst further research is needed, interventions and risk assessments might consider specialised content escalation patterns as a potential, early warning sign of content escalation to CSAM. The findings also suggest that frequency-based models of pornography use may be insufficient on their own: arousal-independent content escalation and the qualitative nature of content may be important for understanding risk trajectories. Future research should employ longitudinal and multimodal methodologies to explore causal mechanisms and situational influences. Examining motivational and inhibitory processes will be key to distinguishing between general compulsive use and specialised content escalation. Additionally, the role of illegality itself as a motivational factor warrants investigation, as some individuals may pursue content primarily for thrill or boundary-crossing rather than content themes (Steely et al., 2018). Finally, research must include more female and gender-diverse samples in pornography and CSAM research to clarify potential differences in escalation patterns.

### Limitations and Future Directions

This study relied on cross-sectional, self-report data, which may be subject to recall bias and social desirability effects, particularly regarding CSAM engagement. Low base rates of illegal content engagement limit statistical power and generalisability. Future studies should incorporate longitudinal designs, experimental paradigms, and objective measures of content engagement. Our sample size was modest, particularly for a principal components analysis and multiple regression. Future studies should include larger samples to examine if the factor structure we identified, and the predictors can be replicated. Additionally, the complex interplay between social, cognitive and situational factors warrants further investigation, particularly regarding peer influences, cognitive rationalisations, and motivational mechanisms.

### Conclusion

In summary, pornography content escalation may be best understood as a multidimensional, non-linear phenomenon. Distinct content escalation clusters appear to reflect different psychological mechanisms, from novelty-seeking and habituation to niche specialisation. Non-Traditional Niche content escalation was associated with CSAM engagement in the present data, providing preliminary support for models that emphasise specialised, boundary-crossing consumption as a possible pathway. These findings offer initial theoretical insights, and directions for future research, but should be interpreted with caution given the cross-sectional design.
